# Artificial intelligence-assisted academic writing: recommendations for ethical use

**DOI:** 10.1186/s41077-025-00350-6

**Published:** 2025-04-18

**Authors:** Adam Cheng, Aaron Calhoun, Gabriel Reedy

**Affiliations:** 1 Departments of Pediatrics and Emergency Medicine, Alberta Children’s Hospital, Cumming School of Medicine, University of Calgary, 28 Oki Drive NW, Calgary, Alberta T3B 6A8 Canada; 2 University of Louisville School of Medicine and Norton Children’s Medical Group, Louisville, KY USA; 3 Faculty of Life Sciences and Medicine, King’s College London, London, UK

**Keywords:** Artificial intelligence, Large language models, ChatGPT, Academic writing, Ethics

## Abstract

Generative artificial intelligence (AI) tools have been selectively adopted across the academic community to help researchers complete tasks in a more efficient manner. The widespread release of the Chat Generative Pre-trained Transformer (ChatGPT) platform in 2022 has made these tools more accessible to scholars around the world. Despite their tremendous potential, studies have uncovered that large language model (LLM)-based generative AI tools have issues with plagiarism, AI hallucinations, and inaccurate or fabricated references. This raises legitimate concern about the utility, accuracy, and integrity of AI when used to write academic manuscripts. Currently, there is little clear guidance for healthcare simulation scholars outlining the ways that generative AI could be used to legitimately support the production of academic literature. In this paper, we discuss how widely available, LLM-powered generative AI tools (e.g. ChatGPT) can help in the academic writing process. We first explore how academic publishers are positioning the use of generative AI tools and then describe potential issues with using these tools in the academic writing process. Finally, we discuss three categories of specific ways generative AI tools can be used in an ethically sound manner and offer four key principles that can help guide researchers to produce high-quality research outputs with the highest of academic integrity.

## Introduction

Generative artificial intelligence (AI) tools emerged into the consumer sector—and into the hands of healthcare professionals, educators, academics and researchers—in late 2022 with the widespread release of the web-based Chat Generative Pre-trained Transformer (ChatGPT) platform [1]. ChatGPT represents a category of generative AI known as large language models (LLMs), which are indexed catalogues of text pulled from human-generated content, designed to build coherent responses to increasingly complex questions based on the statistical relationships inherent in their training datasets [2]. While initial versions of these tools generated relatively low-quality content, this has changed rapidly and continues to improve [3]. Current and future generative AI tools seem to hold great potential for researchers in ways that parallel how other software tools have changed research practice in the past decades [1].

Previous technologies and software tools have helped researchers at every stage of the research process, whether undertaking qualitative or quantitative work. From organising and conducting structured knowledge syntheses and literature reviews, to generating data in new ways, to conducting complex statistical analyses, to analysing highly heterogenous unstructured qualitative data, there is no aspect of the research process that has not been impacted by technological advancements during the first quarter of the twentieth century [4]. Similarly, artificial intelligence features are already being integrated into tools used across the research process, from transcription systems to data analysis software [1, 4].

Unlike these previous tools, however, generative AI tools have introduced a challenge to traditional notions of academic integrity: rather than simply helping researchers more efficiently conduct or complete research tasks, generative AI tools now have the ability to produce novel written content on their own [5–8]. While some researchers have embraced generative AI, others have assiduously avoided engaging with the tools due to a concern that they represent a threat to academic integrity. Researchers, especially academic writers, are then left with a quandary: how can they thoughtfully and carefully engage with generative AI tools in their work while adhering to standards of academic integrity?

As researchers, academic authors, and journal editors, we sought to explore this question for ourselves and realised that there was little clear guidance about the ways that generative AI could be used to legitimately support the production of scientific and academic literature. There has been a notable paucity of research on this topic within the field of healthcare simulation. As a result, healthcare simulation scholars may not fully appreciate the scholarly benefits and scope of ethical risks associated with using LLM-powered generative AI tools for academic writing. This paper provides an overview of the challenges with AI-assisted writing so that all simulation scholars, regardless of the stage in their careers, can responsibly and ethically engage with generative AI tools when producing academic literature.

In this paper, we focus our thinking on how widely available, LLM-powered generative AI tools (e.g. ChatGPT) can help in the academic writing process, and on the underpinning principles that can help guide healthcare simulation scholars to produce high-quality research outputs with the highest of academic integrity. We argue that it is possible to use generative AI tools to support the academic writing process, and that doing so is a legitimate and natural part of the evolution of the global healthcare simulation research community, as long as certain ethical safeguards are adhered to. We first review the ways in which academic publishers are positioning the use of generative AI tools and then explore some of the potential pitfalls of using the tools without consideration of the potential impact they can have on academic output. Using ChatGPT’s own output as a starting point, we finally present an argument for the specific ways in which generative AI can make a legitimate and ethical contribution to academic writing, and provide a series of principles to guide academic writers in the use of these tools.

## What do the journals and publishers say?

To better understand the scope of recommendations provided by journals and publishers about the use of generative AI in academic writing, we conducted a literature search and also asked ChatGPT4 the question: “What recommendations exist for ethical use of ChatGPT in academic writing?” Content from the literature search and ChatGPT response was reviewed, original source materials were identified and discussed, and relevant content was edited and incorporated into this paper along with original ideas and insights from our authorship team.

Academic journals and their publishers have a vested interest in ensuring the integrity of the research that they publish and so explicitly publish their own policies on academic integrity and ethical practice in academic publishing. Further, most academic publishers are voluntary members of the Committee on Publication Ethics (COPE), which helps to establish and support high standards of ethical conduct and reporting of academic research. These principles are clearly applicable to academic writers in the age of generative AI [9]. Further, and especially relevant to the work of academic writers in healthcare simulation, the International Committee of Medical Journal Editors [10], JAMA Network Journals [7], and the World Association of Medical Editors [11] have all issued specific guidelines on the use of generative AI.

Across these statements, some key principles about the use of generative AI resonate. Firstly, and perhaps most importantly, transparency about the use of generative AI tools in the academic writing process is a cornerstone of academic integrity [7, 9, 11]. While specific journals may provide different guidelines of how to acknowledge the use of AI tools, the underlying need for transparent reporting remains paramount. We argue that the most helpful and transparent means for describing the use of these tools is within the methods section of a manuscript, where authors are encouraged to outline how AI tools were utilised and how AI-generated output was handled (e.g. Was the AI-generated text reviewed or discussed? Was the text edited? When relevant, were original sources of information identified?) and incorporated into the final manuscript. The core elements of this disclosure may change over time with the rapidly evolving landscape of generative AI. Listing the use of generative AI in an acknowledgements section of a manuscript is another possibility, according to some journals, but these are often at the end of a manuscript and hence less likely to be seen by readers. More explicit foregrounding of the use of generative AI in the methods section of a manuscript avoids any transparency concerns.

Another core principle reflected in these statements is that, despite their ability to generate novel and new text, generative AI tools do not meet the generally agreed standards of authorship of academic research [7, 9, 11, 12]. Scientific authorship reflects both contribution to the work and accountability for the work. As a software tool, a generative AI platform can produce written content that, in theory, could be included as a contribution to a manuscript. These tools, however, cannot be held responsible for the accuracy of the manuscript’s content, nor approve a submitted or published manuscript or vouch to support any subsequent investigation of claims made against the work. For these reasons, generative AI tools are not considered authors of academic work and should not be credited as such. Across the statements, another idea that emerges is the primacy of the creative and intellectual contributions of the authors of academic work [7, 9, 11]. While generative AI tools can help to support and improve the academic writing process, they must not be used in place of the contributions of the academic research team.

## Issues with AI-assisted writing

The potential benefits of incorporating LLM-based generative AI tools into the academic manuscript writing process has led to studies exploring its capabilities. As these generative AI tools (e.g. ChatGPT) generally source data from publicly available internet content [5], studies have uncovered important issues which raise legitimate concern about the utility, accuracy, and integrity of AI in writing medical manuscripts. Studies highlight three key challenges with content created by LLM-based generative AI tools: (a) plagiarism [5, 6]; (b) fabricated or false information (i.e. ‘AI hallucination’) [13–15]; and (c) inaccurate or fabricated references [13–17].

Generative AI tools access publicly available data to generate text, which may produce content that closely (or exactly) resembles the original source of information [6]. Similarities in content that are undetected and unedited by researchers raises concern for plagiarism and potential copyright infringement (e.g. use of published figure without attribution or permission) [18]. Without appropriate content expertise, some authors may be unaware of AI-generated text that is plagiarised. Left unchecked, this may result in a snowball effect, where authors unknowingly cite AI-generated plagiarised text published in other articles (i.e. double plagiarism) [5].

‘AI Hallucination’ is a phenomenon where the generative AI tool offers convincing, but completely fabricated, nonsensical, or factually inaccurate content in response to prompts provided by the user [13–15]. As generative AI tools are not designed to assess accuracy or authenticity of content, they are prone to produce fabricated content when trained on large amounts of unsupervised data, as is the case of ChatGPT. While new models of ChatGPT have ‘learned’ compared to prior releases, they cannot differentiate between real and factitious data that is received, integrated and used to generate new content [19]. Further complicating this issue is generative AI’s limited ability to comprehend complex commands [14]. Consequently, when asked to write medical abstracts, case reports, or research proposals, these generative AI tools frequently include inaccurate statements or fabricated content fueled by false sources of data [13–15]. A final issue is that AI tools will tend to generate output that reproduces or amplifies biases inherent within the source data [19]. These troublesome patterns may lead to the spread of misinformation or bias, or threaten researcher integrity if left unchecked [5].

LLM-powered generative AI tools are notoriously poor at referencing medical literature. Athaluri et al. tasked ChatGPT to write 50 research protocols with references on a spectrum of novel medical research topics [15]. Amongst the references generated for these research protocols, 38% had the wrong DOI or a fabricated DOI, and 16% of the referenced articles were completely fabricated, suggesting that the ability of ChatGPT to generate accurate references is potentially linked to the availability of a DOI and accessibility of online articles [15]. Another study found that amongst 30 short medical papers generated by ChatGPT, nearly half of the references were fabricated, while 46% of the references were authentic but inaccurate [16]. Amongst the inaccurate references, 48% were errors with the title, 52% represented errors with authorship, and 93% of these references had the wrong PMID. Only 7% of all the references generated by ChatGPT were completely authentic and accurate, signalling a significant reliability issue when it comes to ChatGPT’s ability to generate accurate references [16].

Rapid technological changes may render these issues insignificant over time, as has been demonstrated with the evolution of generative AI tools to date. Researchers ultimately hold full responsibility for the originality of their manuscript, accuracy and relevance of content, and appropriate referencing of published literature. As a result, we caution against the sole use of LLM-based generative AI to (1) write content that will be used verbatim (i.e. without human editing) in medical abstracts, articles, or research proposals; and (2) generate references that will be used in medical abstracts, articles, or research proposals. Even if authors double check content for accuracy, relevance, reasoning, bias, and appropriate referencing, they are still assuming some inherent risk given the issues described above.

**Ethical use of AI for writing.**

**Fig. 1:**
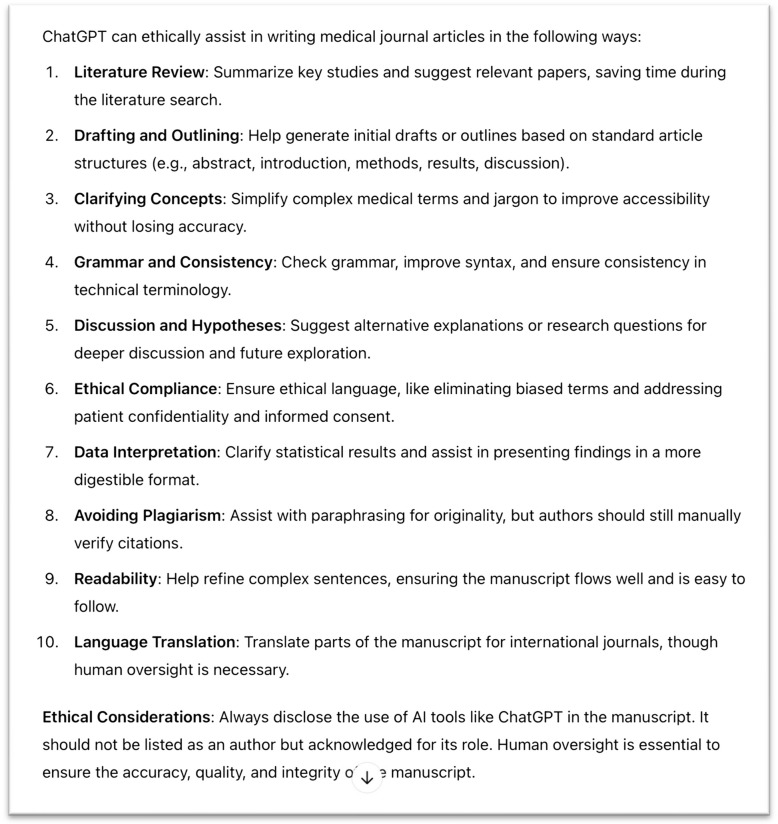
ChatGPT 4o response to the prompt: ‘Can you please provide a list of ways ChatGPT can be ethically used to assist authors in writing articles for medical journals?’

**Fig. 2:**
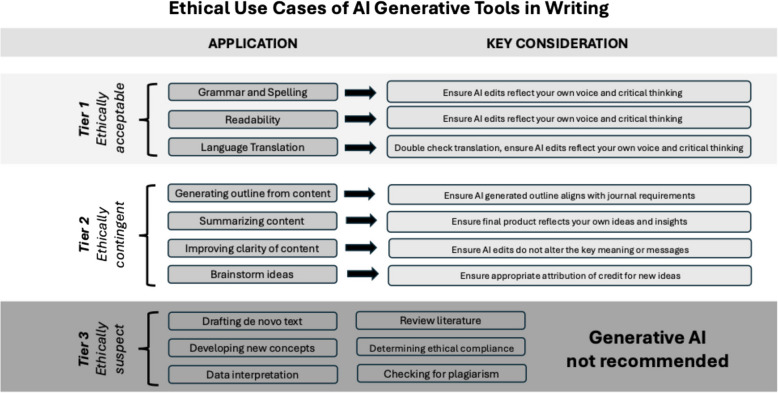
Ethical use of AI generative tools in writing

In our effort to define how generative AI tools can be ethically used to assist in writing for healthcare and related academic journals, we asked ChatGPT 4.0 the following question: ‘Can you please provide a list of ways ChatGPT can be ethically used to assist authors in writing articles for medical journals?’ The results of this request are displayed in Fig. 1. We found that ChatGPT oversimplified many items in this list, which may results in misleading guidance for academic authors and scholars. We collectively reflected on the recommendations made by ChatGPT, discussed our personal insights, and considered existing research on this topic before deciding which items should be included, modified or excluded. The resultant Fig. 2 provides our suggestions on how LLM-based generative AI can be ethically used to support the academic writing process.

When analysing these suggestions, it is helpful to consider two questions. First, “What exactly are LLM’s such as ChatGPT trained to do?”. In terms of LLM training, specifically, it is important to understand that systems such as these represent mathematical models mapping specific segments of text and symbols (often called ‘tokens’) in the initial request with segments of text and symbols in the LLM’s ‘answer’ [2]. This mapping process utilises an extensive set of pre-learned token associations derived from large sets of human-generated data (typically the internet). While the ‘meaning’ of these tokens is implicit to some extent within these associations, the model is not explicitly utilising this meaning, but is rather generating syntactically appropriate strings of text based on statistical associations [20]. There are no ‘fact-checking’ components to this process in current models, which is an important source of the AI hallucinations and propagation of biases within the training dataset. Given this, we find it helpful to view LLM’s functionality as the creation of syntactically appropriate text that expresses associations already present within the training data, with the understanding that this text may or may not express ‘accurate’ data, and that this text may be subject to implicit or explicit bias [2]. The potentially helpful and ethical uses of ChatGPT for academic writers, then, will be those that utilise its strengths with structure and syntax while avoiding its weaknesses via human oversight and review.

The second question warranting consideration is “Will the specific use of ChatGPT have a deleterious effect on human critical thinking and scholarly development?” As researchers, it is vital that we be concerned with more than just productivity, but with the professional growth and evolution of scholars within our field. This includes assuring that they are able to think deeply and creatively about both the research problems they are investigating, and their resulting data. While novice scholars are in most need of developing these skills, even mature researchers benefit from adapting their research skills to the evolving landscape of healthcare simulation research. It is a concern, then, if scholars become too dependent on ChatGPT for ideation, generation of primary written content, and initial data interpretation, as this assistance could easily devolve into a dependency that arrests further scholarly development.

Using this heuristic, we organised the items on the list provided by ChatGPT into ‘ethical tiers’ (Fig. 2). The most *ethically acceptable* tier includes those items in which ChatGPT is primarily used to re-structure previously existing text or ideas, including grammar and spelling, readability, and language translation. While ChatGPT is not designed to directly check grammar and spelling, it will respond to prompts by rewriting content in a grammatically correct fashion without spelling mistakes. It will also help with refining the readability and flow of the manuscript, assuring that it uses the best grammatical syntax. Authors should always double check the refined text to ensure the edits still closely reflect their own voice and critical thinking, and that it is free of grammatical errors and spelling mistakes. Authors looking for real-time writing support (e.g. grammar and spelling suggestions as you write), proactive suggestions to rewrite sentences, and adjusting the tone of writing should look to AI writing assistants (e.g. Grammarly), which have been specifically designed for these purposes [21]. LLM-powered generative AI tools can also be used to provide translation of text into a semantically equivalent receptor language, which may be helpful for researchers looking to submit an article in a non-native language. However, some limitations are that ChatGPT is unable to accurately interpret the subtle nuances between languages, slang words, or cultural terms, which sometimes leads to inaccurate translation [22]. Researchers using ChatGPT to translate academic manuscripts should always have a human author (ideally a native speaker of the receptor language) review the final manuscript for accurate translation. These and similar uses best fit the model’s strengths while not encouraging over-reliance on the tool for tasks requiring critical thought.

The next tier represents a more *ethically contingent* group of possibilities, as their appropriate use depends primarily on the steps the author takes when working with AI-generated content. Items in this tier include the use of ChatGPT for generating an outline, summarising content, improving clarity of drafted content, or brainstorming ideas. In each case, these uses task ChatGPT with generating novel text and thus have heightened potential for the introduction of bias, hallucination, or plagiarism if engaged in uncritically. ChatGPT is most skilled and accurate at text manipulation when the necessary ideas are already present in the initial query. Thus, using ChatGPT to generate an outline from a prompt containing manuscript content, or transforming a roughly outlined portion of text that contains all necessary concepts into a more powerfully written version may be of potential value. Refining a previously constructed outline containing all core concepts or expanding a clearly specified concept into a more elaborate summary may also represent ethical use of generative AI. In all such use cases, however, authors should ensure the final product accurately reflects their own ideas and insights and that the AI-generated content did not alter the key meaning or message. Finally, asking ChatGPT to brainstorm ideas based on a prompt containing questions or original text may also provide some benefit. For example, authors may ask ChatGPT to offer arguments or counter-arguments for a certain philosophical position or viewpoint, which can then be helpful to expand perspectives when crafting a discussion. When used to brainstorm ideas, authors must be certain these ChatGPT-generated ideas are attributed and edited appropriately to ensure accuracy and to eliminate or reduce the risk of plagiarism.

Asking generative AI to do slightly more than the above, however, may make application of ChatGPT in these use cases *ethically suspect* our final tier of uses. For example, drafting de novo text without providing original content in the prompt or developing new concepts for a particular section of text risks not only introducing factually incorrect material but also deprives the authors of an opportunity to engage deeply with the source material on which their paper is based on a personal level. Such deep engagement is vital for developing a comprehensive understanding of the research question. Other use cases fitting within this final tier includes the use of ChatGPT for data interpretation, literature review, and checking for ethical compliance and plagiarism. The use of ChatGPT to perform the primary analysis of study data introduces the possibility of bias and hallucination and effectively short-circuits an intellectual process that is most properly the domain of the authors. We believe that this task is best left for the authors themselves as, by analysing the data in its entirely first, they gain a fuller understanding of the results which can then be used to critique any interpretations inserted by Chat GPT.

As noted above, published evidence indicates that ChatGPT is notoriously unreliable in citing references and can easily generate grammatically perfect references that do not actually exist. While some specialist products designed to support literature reviews are emerging (e.g. Elicit), any literature search performed using ChatGPT must thus be confirmed with a traditional search engine, thus negating the actual benefit of using generative AI. The use of ChatGPT to assure ethical, unbiased language is even more suspect, due to unavoidable biases contained within the textual databases that are used to train the LLM statistical models. Barring future developments that permit the creation of truly unbiased training datasets, there is a reasonable chance that the indiscriminate use of ChatGPT in this way will introduce and perpetuate, rather than remove, bias. Training set issues also form the basis of our concerns regarding the use of AI to avoid plagiarism, as it is possible to query LLM’s in a manner that causes them to reproduce elements of their training set verbatim, thus potentially introducing plagiarised text that otherwise would not find its way into the manuscript [18]. Determining the best language to use to assure neutrality and objectivity, and the avoidance of plagiarism, is thus best left to the human authors.

## Author checklist for ethical use of AI generative tools

**Fig. 3:**
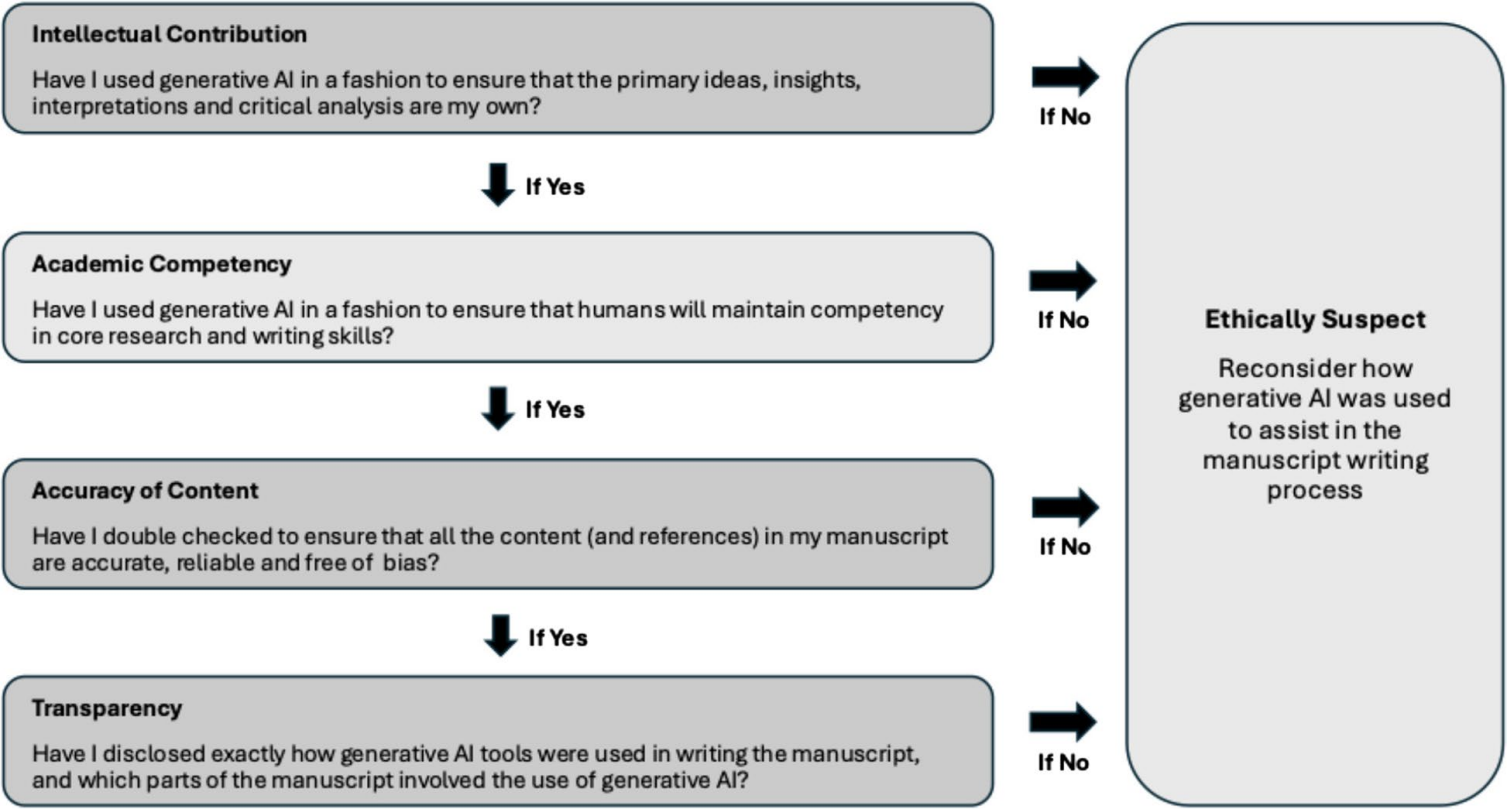
Author checklist—key considerations for the ethical use of generative AI tools in manuscript writing

The importance of the above exercise is not that it provides an exhaustive list of ethically permissible and impermissible actions. Rather, it showcases the value of taking a careful, nuanced approach to thinking through these issues. Mann et al. recently suggested three criteria for responsible use of LLMs in scholarship [23]: (1) Human vetting and guaranteeing; (2) Substantial human contribution; and (3) Acknowledgement and transparency. We agree with these core principles and look to build on these further by encouraging authors to reflect on a series of four questions to guide their use of generative AI in the manuscript writing process (Fig. 3): (1) Have I used generative AI in a fashion to ensure that the primary ideas, insights, interpretations, and critical analyses my own? (2) Have I used generative AI in a fashion to ensure that humans will maintain competency in core research and writing skills? (3) Have I double checked to ensure that all the content (and references) in my manuscript are accurate, reliable, and free of bias? and (4) Have I disclosed exactly how generative AI tools were used in writing the manuscript, and which parts of the manuscript involved the use of generative AI? If the answer to any of these questions is no, then we strongly encourage the author to reflect on the writing process and reconsider steps to directly address the ethical issue. And, even if the answers to questions 1 to 3 suggest that generative AI use is ethical, it is still necessary to disclose it clearly, explicitly and transparently within the article, in a manner similar to other technological tools (such as statistical programmes) that are used.

## Summary and future directions

As a final note, the concepts discussed in this paper apply solely to ChatGPT and other similar LLM-powered generative AI tools. Artificial intelligence is a broad and rapidly evolving set of technologies. As the technology evolves, so will recommendations for ethical use. We hope that the principles we have outlined will remain salient when considering how to ethically engage with these tools as they develop, but revision may be needed. Furthermore, we have not discussed generative AI tools developed specifically for the academic community, such as Scopus AI, which draws data from abstracts, author profiles, articles, books, reviews, and other reliable sources [24]. Given that the data sources of academic generative AI tools are notably more reliable and robust, it is possible that the issues of bias and hallucinations may be mitigated in these platforms. Future research should explore the ethical use of academic generative AI tools for supporting the writing process.

The caution regarding diminishment of human capacity cannot be dispensed with so quickly. Above all, our goal is to further our field by training and promoting researchers able to think deeply about the questions we face and offer cogent solutions based on evidence. Doing this requires researchers who develop real, deep knowledge about the subjects they investigate and who are able to reason clearly regarding their work. Outsourcing some of these intellectual tasks to artificial tools runs the risk of these necessary skills being lost over time. Indeed, the real challenge is defining when and how to optimally use generative AI, and how to ethically manage the nuances of using AI in the academic writing process. Continued discourse within the healthcare simulation community will be essential to ensure that the ethical application of generative AI tools in academic writing is consistent with the evolutionary state of the technology over time.

## Data Availability

No datasets were generated or analysed during the current study.

## Abbreviations

AI: Artificial Intelligence
ChatGPT: Chat Generative Pre-trained Transformer
COPE: Committee on Publication Ethics
LLMs: Large language models

## References

[1] Xu X, Chen Y, Miao J. Opportunities, challenges, and future directions of large language models, including ChatGPT in medical education: a systematic scoping review. Journal of educational evaluation for health professions. 2024;21:6. 38486402 10.3352/jeehp.2024.21.6 PMC11035906

[2] Thirunavukarasu AJ, Ting DSJ, Elangovan K, Gutierrez L, Tan TF, Ting DSW. Large language models in medicine. Nat Med. 2023;29(8):1930–40. 37460753 10.1038/s41591-023-02448-8

[3] Kim S-J. Trends in research on ChatGPT and adoption-related issues discussed in articles: a narrative review. Science Editing. 2023;11(1):3–11.

[4] Khalifa M, Albadawy M. Using artificial intelligence in academic writing and research: an essential productivity tool. Computer Methods and Programs in Biomedicine Update.2024;5:100145.

[5] Kim S-J. Research ethics and issues regarding the use of ChatGPT-like artificial intelligence platforms by authors and reviewers: a narrative review. Science Editing. 2024;11(2):96–106.

[6] Kacena MA, Plotkin LI, Fehrenbacher JC. The use of artificial intelligence in writing scientific review articles. Curr Osteoporos Rep. 2024;22(1):115–21. 38227177 10.1007/s11914-023-00852-0 PMC10912250

[7] Flanagin A, Bibbins-Domingo K, Berkwits M, Christiansen SL. Nonhuman “Authors” and implications for the integrity of scientific publication and medical knowledge. JAMA. 2023;329(8):637–9. 36719674 10.1001/jama.2023.1344

[8] Dergaa I, Chamari K, Zmijewski P, Ben SH. From human writing to artificial intelligence generated text: examining the prospects and potential threats of ChatGPT in academic writing. Biol Sport. 2023;40(2):615–22. 37077800 10.5114/biolsport.2023.125623 PMC10108763

[9] COPE position - Authorship and AI - English. Available from: https://publicationethics.org/guidance/cope-position/authorship-and-ai-tools. Accessed December 1, 2024.

[10] Editors ICoMJ. Recommendations for the conduct, reporting, editing, and publication of scholarly work in medical journals 2025. Available from: https://www.icmje.org/icmje-recommendations.pdf. Accessed April 4, 2025. 25558501

[11] Zielinski C, Winker MA, Aggarwal R, Ferris LE, Heinemann M, Lapena JF Jr, et al. Chatbots, generative AI, and scholarly manuscripts: WAME recommendations on chatbots and generative artificial intelligence in relation to scholarly publications. Colomb Med (Cali). 2023;54(3): e1015868. 38089825 10.25100/cm.v54i3.5868 PMC10712422

[12] McNutt MK, Bradford M, Drazen JM, Hanson B, Howard B, Jamieson KH, et al. Transparency in authors’ contributions and responsibilities to promote integrity in scientific publication. Proc Natl Acad Sci U S A. 2018;115(11):2557–60. 29487213 10.1073/pnas.1715374115 PMC5856527

[13] Alkaissi H, McFarlane SI. Artificial hallucinations in ChatGPT: implications in scientific writing. Cureus. 2023;15(2): e35179. 36811129 10.7759/cureus.35179 PMC9939079

[14] Buholayka M, Zouabi R, Tadinada A. The readiness of ChatGPT to write scientific case reports independently: a comparative evaluation between human and artificial intelligence. Cureus. 2023;15(5): e39386. 37378091 10.7759/cureus.39386 PMC10292135

[15] Athaluri SA, Manthena SV, Kesapragada V, Yarlagadda V, Dave T, Duddumpudi RTS. Exploring the boundaries of reality: investigating the phenomenon of artificial intelligence hallucination in scientific writing through ChatGPT references. Cureus. 2023;15(4): e37432. 37182055 10.7759/cureus.37432 PMC10173677

[16] Bhattacharyya M, Miller VM, Bhattacharyya D, Miller LE. High rates of fabricated and inaccurate references in ChatGPT-generated medical content. Cureus. 2023;15(5): e39238. 37337480 10.7759/cureus.39238 PMC10277170

[17] Benichou L, ChatGpt. The role of using ChatGPT AI in writing medical scientific articles. J Stomatol Oral Maxillofac Surg. 2023;124(5):101456. 10.1016/j.jormas.2023.101456 36966950

[18] The plagiarism problem: how generative ai models reproduce copyrighted content. Available from: https://bardai.ai/2024/01/10/the-plagiarism-problem-how-generative-ai-models-reproduce-copyrighted-content/. Accessed December 1, 2024.

[19] Hosseini M, Rasmussen LM, Resnik DB. Using AI to write scholarly publications. Account Res. 2024;31(7):715–23. 36697395 10.1080/08989621.2023.2168535 PMC10366336

[20] Ferrer J. How transformers work: a detailed exploration of transformer architecture 2024. Available from: https://www.datacamp.com/tutorial/how-transformers-work?utm_source=google&utm_medium=paid_search&utm_campaignid=19589720830&utm_adgroupid=157156377071&utm_device=m&utm_keyword=&utm_matchtype=&utm_network=g&utm_adpostion=&utm_creative=684592141199&utm_targetid=dsa-2218886984380&utm_loc_interest_ms=&utm_loc_physical_ms=9191512&utm_content=dsa~tofu~tutorial~machine-learning&accountid=9624585688&utm_campaign=230119_1-sea~dsa~tofu_2-b2c_3-nam_4-prc_5-na_6-na_7-le_8-pdsh-go_9-nb-e_10-na_11-na&gad_source=1. Accessed April 4, 2025.

[21] Grammarly vs. ChatGPT: choosing the right AI writing assistant. Available from: https://www.grammarly.com/blog/grammarly-vs-competition/grammarly-vs-chatgpt/#:~:text=Here's%20an%20example%20of%20Grammarly's,Writing%20Coach%2C%20to%20get%20feedback. Accessed December 1, 2024.

[22] How good is ChatGPT at translating? Available from: https://www.amperetranslations.com/blog/chatgpt-translation/. Accessed December 1, 2024.

[23] Mann SP, Vazirani AA, Mateo A, Earp BD, Minssen T, Cohen IG, et al. Guidelines for ethical use and acknowledgement of large language models in academic writing. Nature Machine Intelligence. 2024;6:1272–4.

[24] Scopus AI: Trusted content. Powered by responsible AI. Available from: https://www.elsevier.com/products/scopus/scopus-ai. Accessed December 1, 2024.

